# Effects of gender affirming hormone therapy with testosterone on renal function of assigned female at birth transgender people: a meta-analysis

**DOI:** 10.3389/fendo.2025.1537838

**Published:** 2025-06-12

**Authors:** Daniele Tienforti, Luca Spagnolo, Luca Piscitani, Camilla Tonni, Vittoria Donatelli, Giuliana Cordeschi, Marco Giorgio Baroni, Arcangelo Barbonetti

**Affiliations:** ^1^ Andrology Unit, Department of Clinical Medicine, Life, Health and Environmental Sciences, University of L’Aquila, L’Aquila, Italy; ^2^ Spinal Unit, San Raffaele Sulmona Institute, Sulmona, Italy; ^3^ Nephrology and Dialysis Division, Department of Medicine, San Salvatore Hospital, L’Aquila, Italy; ^4^ Faculty of Bioscience and Technology for Food, Agriculture and Environment, University of Teramo, Teramo, Italy

**Keywords:** AFAB, testosterone, creatinine, gender dysphoria, gender incongruence, kidney

## Abstract

**Objective:**

The impact of testosterone-based gender affirming hormone therapy (T-GAHT) on kidney function in transgender individuals assigned female at birth (AFAB) remains a matter of clinical uncertainty and debate. This study aimed to quantify through a meta-analytical approach the changes in estimated glomerular filtration rate (eGFR), a widely used clinical parameter that reflects how efficiently the kidneys filter waste products from the blood, and in secondary markers of kidney functions in this population during 24 months of GAHT. The eGFR was calculated using the CKD-EPI (Chronic Kidney Disease Epidemiology Collaboration) equation, which estimates kidney filtration based on serum creatinine, age, and sex.

**Methods:**

A thorough search of MEDLINE, COCHRANE LIBRARY, SCOPUS and WEB OF SCIENCE databases was carried out to identify suitable studies. Quality of the articles was scored using the Effective Public Health Practice Project. Data were combined using random effects models and the between-study heterogeneity was assessed using Cochrane’s Q and I^2^.

**Results:**

Twenty included studies provided information about an overall sample of 2380 individuals. The pooled estimates documented a significant decrease in eGFR (CKD-EPI equation) at 6 and 12 months with respect to baseline, using the attributed (female) gender. When the CKD-EPI equation was referred to the perceived (male) gender, eGFR significantly decreased after 12 months but not after 6 months of T-GAHT. The trend of eGFR values showed a transient decline during the first year of therapy, followed by stabilization at 18 and 24 months. This pattern is likely attributable to increased creatinine production due to testosterone-induced gains in muscle mass, rather than to a true decline in kidney function. Among the secondary outcomes, pooled estimates revealed significant increases of creatinine and uric acid levels at all follow-up times. On the contrary, blood urea nitrogen (BUN), a waste product filtered by the kidneys and commonly used to assess renal function, did not change significantly after either 6 months or 12 months of T-GAHT.

**Conclusions:**

The influence of T-GAHT on eGFR in the first two years in healthy, young AFAB transgender individuals appears to be statistically significant, but is likely not clinically relevant. This interpretation is supported by the stability of BUN levels and the absence of adverse renal events in the included studies, suggesting preserved kidney function despite changes in creatinine-based estimates. Further research is warranted to identify more accurate tools for evaluating kidney function in this population, particularly during the early months of treatment or in individuals with pre-existing renal conditions.

**Systematic review registration:**

https://www.crd.york.ac.uk/prospero/, identifier CRD42024596106.

## Introduction

Interpretation of laboratory tests in transgender individuals can be challenging in routine care, especially when the analytes have sex-specific reference intervals (1, 2). Health care professionals may be asked whether to use reference ranges based on assigned sex, self-identified gender, or a combined approach (3). Factors such as the type of hormone therapy initiated or its duration may influence this decision (4). Accurate interpretation is crucial for appropriate clinical decision-making.

This issue becomes particularly relevant when assessing the effects of gender-affirming hormone therapy (GAHT) on kidney function, a parameter influenced by several clinical and lifestyle-related factors (e.g., hydration, nutrition, comorbidities, and medications) and monitored through various biochemical markers. Available evidence does not clearly establish whether, and to what extent, testosterone preparations affect renal function in cisgender individuals (5), nor whether GAHT may exacerbate pre-existing renal impairment in transgender people assigned female at birth (AFAB) (6, 7).

Indeed, the assessment of kidney function in this population undergoing testosterone-based GAHT (T-GAHT) presents several challenges. First, testosterone treatment, by increasing muscle mass—and consequently serum creatinine (SCr) levels—could lead to an overestimation of renal dysfunction or may lead to a misclassification of kidney function (8). Second, there is still no specific formula validated for estimating glomerular filtration rate (eGFR) in transgender individuals. Currently, the most widely used formula is the Chronic Kidney Disease Epidemiology Collaboration (CKD-EPI) equation (9), which incorporates sex as a covariate because creatinine production is lower in cisgender females than in cisgender males due to differences in muscle mass; in addition, substantial sex- and gender-related differences in diet may exist. Consequently, if these factors are not properly accounted for, eGFR may be systematically overestimated in cisgender females (10).

In an attempt to overcome this limitation, some researchers suggest calculating an “intermediate” GFR value by averaging male and female estimates (11), while others recommend using the sex corresponding to the individual’s gender identity if GAHT has been ongoing for more than six months, although this approach is better validated for transgender men (12).

While these methodological challenges are important and deserve careful consideration, the central clinical question remains whether testosterone-based GAHT itself induces measurable changes in renal function in AFAB individuals. To address this question, we conducted a comprehensive systematic review and meta-analysis aimed at evaluating whether T-GAHT leads to clinically meaningful alterations in renal parameters—including eGFR, SCr, uric acid, and blood urea nitrogen (BUN)—over a follow-up period of up to 24 months. By doing so, we aimed to contribute to the expanding field of transgender medicine by offering a clearer understanding of the renal effects of masculinizing hormone therapy, beyond the technical difficulties inherent to laboratory test interpretation.

## Methods

The study was conducted according to the statement Preferred Reporting Items for Systematic reviews and Meta-analyses protocols (PRISMA-P) (13); it also complies with the guidelines for Meta-Analyses and Systematic Reviews of Observational Studies (MOOSE) (14). The study protocol was registered in the international prospective registry for systematic reviews (PROSPERO) with registration number CRD42024596106.

### Systematic search strategy

A systematic search was carried out in PubMed, Scopus, Web of Science and Cochrane Library to identify the studies published in English on this topic up to November 2024. The databases were queried by means of a purpose-built search string using the biomedical vocabulary Medical Subject Headings (MeSH) of PubMed. For the extraction of publications (records), the following terms were used: “transgender”, “AFAB”, “FtM”, “female to male”, “transmen”, “trans men”, “GAHT”, “gender-affirming hormone therapy”, “testosterone”, “androgen*”, “kidney”, “uric acid”, “BUN”, “urate”, “urea”, “creatinine”, “proteinuria”, “cystatin”, “creatinine clearance”, eGFR, GFR, “Cockcroft-Gault”, “MDRD”, “CKD-EPI”, “SCr/Q”, “CKiDU25”. To combine these key terms we used the Boolean operators AND/OR. If it was not clear from reading the abstract whether the study contained relevant data, the full text was retrieved. Finally, possible additional studies were identified by means of a manual search among the references cited in the articles included.

### Inclusion and exclusion criteria

The study selection for inclusion was carried out in several stages. In the identification phase, querying the databases identified potentially eligible studies that could be included in the meta-analysis. Following the removal of repeated articles (same publication found in more than one database), in the second phase, studies of possible interest were screened by reading the title and abstract. In the third phase, the remaining articles were assessed in full text for eligibility. The following criteria were used: (1) studies enrolling AFAB transgender individuals undergoing T-GAHT; (2) availability of pre- and post-intervention values related to the primary outcome and/or one or more of the secondary outcomes, as reported below. Observational studies, as well as longitudinal intervention studies were considered eligible, while we excluded studies that did not focus on the target population, lacked relevant outcomes, used a non-eligible design, or presented incomplete or inconsistent data. Two independent reviewers (D.T., L.S.) assessed the full text of all selected studies to establish eligibility, and any disagreements were resolved through an open discussion involving a third reviewer (A.B.). The flow-chart proposed by Page et al. (15) was used to schematize the steps for the inclusion of studies.

### Quality assessment

The methodological quality of the included articles was established using the Quality assessment tool for Quantitative studies developed by the Effective Public Health Practice Project (EPHPP) (16). This quality assessment tool, used for intervention studies as well as randomized controlled and case-control studies, was also validated for systematic reviews (17). It considers the following domains: selection bias, study design, confounding factors, study blindness, data collection method and losses at follow-up. The quality of each domain can be indicated as strong (strong), moderate (moderate) or weak (weak), and in the overall judgment, the quality can be considered strong, if no weak score was assigned, moderate, if only a weak judgment was assigned to one of the domains, and finally, weak, if two or more weak judgments were assigned to several domains. Two independent reviewers (D.T., L.S.) performed the quality assessment.

### Data extraction

Data were extracted from the studies selected by two independent reviewers (D.T., A.B.). The primary outcome was the mean difference in estimated glomerular filtration rate (eGFR) at 6, 12, 18 and 24 months of GAHT with respect to the baseline, using the CKD-EPI equation for both attributed (female) and self-identified (male) gender. The secondary outcomes were the variations over time in SCr, blood urea nitrogen (BUN), uric acid (UA) levels, systolic (SBP) and diastolic blood pressure (DBP). Additional information extracted, when available, were mean age and body mass index (BMI) of the participants, as well as the type of testosterone preparation administered.

### Statistical analysis

The effect of the T-GAHT on kidney parameters was assessed with Mantel-Haenszel estimates using the mean difference (MD) and 95% confidence interval (CI) when different follow-up times were compared with the baseline values. The Cochran’s χ^2^ (Cochran’s Q) and I^2^ tests were carried out to analyze statistical heterogeneity between the results of different studies: I^2^>50% and/or p<0.05 indicated substantial heterogeneity (18, 19). Data were combined using a random effects model. Even when a low heterogeneity was detected, a random-effects model was applied, because the validity of tests for heterogeneity can be limited with a small number of included studies. Publication bias was explored through the funnel plot (20) and Duval and Tweedie trim-and-fill test (21), to help detect presumed missing studies to rebalance the funnel distribution in the presence of a skewed shape. In addition, the test recalculates the combined estimate after the inclusion of these putative missing studies, thus correcting the analysis for publication bias. Data were analyzed using the Review Manager of the Cochrane Library (version5.3; The Nordic Cochrane Centre, The Cochrane Collaboration, Copenhagen, Denmark) and the R statistical software (version 3.6.3, 2020; The R Foundation for Statistical Computing, Vienna, Austria) with the “metafor” package.

## Results

### Study selection

**Figure 1:**
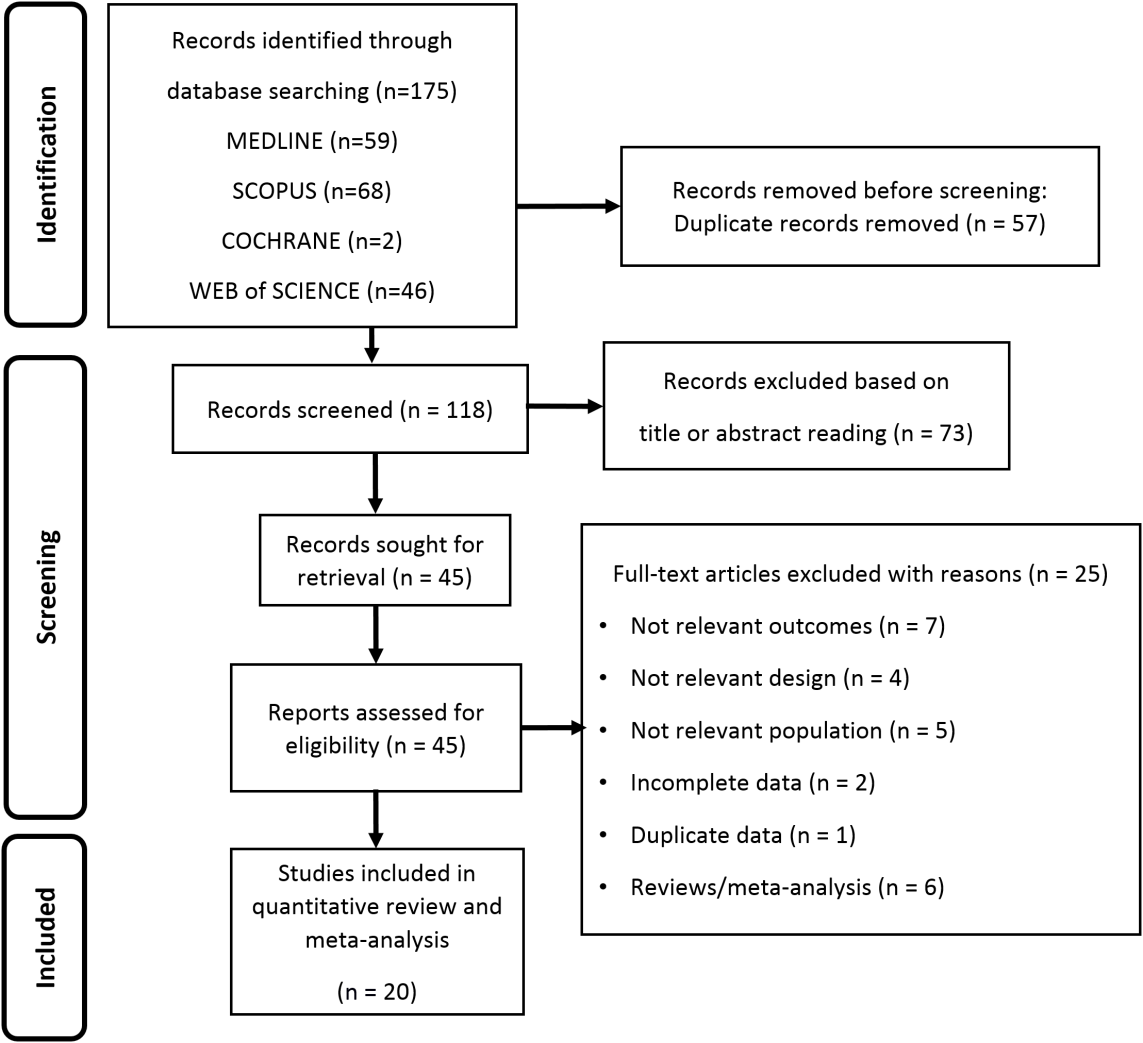
Flow diagram showing an overview of the study selection process.

Searching from database yielded a total of 175 studies. Removal of duplicates resulted in a total of 118 publications, of which 73 were judged to be irrelevant simply by reading the title and abstract. Thus, as shown in **Figure 1**, 45 articles were identified, of which 20 met the inclusion criteria (12, 22–40). Details of the studies included in the quantitative synthesis are summarized in **Table 1**.

**Table 1:** Main characteristics of the included studies.

First author	Year	Country	Study design	N	Mean age (years)	Mean BMI	Testosterone treatment	Parameters	Follow-up visits (months)
Chen (22)	2019	multicentric	prospective	30	24 ± 6	24.5 ± 6	TG 50 mg day MTE 250 mg/2–3 weeks	creatinine	0-3
Fadich (12)	2022	USA	retrospective	29	26 ± 9	29.4 ± 6.8	TG 30.5 mg/day TDP 2–4 mg/24 hour MTE 60 mg/week	creatinine BUN CKD-EPI	0-6-12
Fernandez & Tannock (23)	2016	USA	retrospective	19	30 ± 8	28.1 ± 2.1	TU 11.36 mg/day	creatinine	0-3-6-18
Humble (24)	2019	USA	retrospective	25	24 ± 6	29.6 ± 8.4	NS	creatinine BUN	0-6
Kirisawa (25)	2021	Japan	retrospective	85	27 ± 6	NR	TE 125–250 mg	uric acid	0-3-6-12-24
Korpaisarn (26)	2021	Thailand	retrospective	34	28 ± 6	24.6 ± 4.8	TE 50–100 mg/week	creatinine uric acid	0-24
Kurahashi (27)	2013	Japan	retrospective	160	NS	NR	TE 125–250 mg	uric acid	0-3-6-12
Liu (28)	2020	Taiwan	retrospective	65	28 ± 1	22.6 ± 0.3	NR	creatinine	0-6-12-24
Maheshwari (29)	2022	USA	retrospective	24	25 ± 7	35.8 ± 5.1	NS	creatinine	0-3-6-12
Meriggiola (30)	2008	multicentric	prospective	5	34 ± 4	22.2 ± 2.1	MTE 110 + 25 mg/7–15 days	creatinine BUN	0-12
Millington (31)	2022	USA	prospective	194	16 ± 2	23.9 ± 1.4	TCsc 40 mg/week TDP 40.5 mg/day	creatinine CKD-EPI	0-6-12-18-24
Scharff (32)	2019	multicentric	prospective	278	24 ± 7	25.5 ± 5.6	TG 50 mg day MTE 250 mg/2–3 weeks TU 1000 mg/12 weeks	creatinine	0-12
Stoffers (33)	2019	the Netherlands	retrospective	62	17 ± 3	22.4 ± 3.4	TP 125/2 weeks	creatinine	0-6-12-24
Tominaga (34)	2024	Japan	retrospective	291	25 ± 6	22.1 ± 3.2	TE 62.5-125-250	creatinine uric acid	0-3-6-9-12-24→120
Van Eeghen (35)	2023	the Netherlands	prospective	285	23 ± 1	26.0 ± 6.0	NS	creatinine CKD-EPI	0-12
Van Kesteren (36)	1996	the Netherlands	prospective	19	26 ± 6	21.7 ± 2.4	MTE 250 mg/2–3 weeks	creatinine	0-12
Vlot (37)	2019	the Netherlands	prospective	132	26 ± 9	24.9 ± 1.5	TU 1000 mg/12 weeks TG 50 mg/day MTE 250 mg/2–3 weeks	creatinine	0-12
Wiepjes (38)	2019	the Netherlands	retrospective	543	26 ± 2	25.6 ± 5.7	NS	creatinine	0-12
Wierckx (39)	2014	multicentric	prospective	53	25 ± 8	24.8 ± 5.3	TU 1000 mg/12 weeks MTE 250 mg/2 weeks	creatinine	0-12
Yahyaoui (40)	2008	Spain	prospective	47	25 ± 5	25.4 (4.9)	TDP 5 mg/day MTE 250 mg/2 weeks	uric acid	0-12-24

MTE, mixed testosterone esters; NS, not specified; TT, total testosterone; TC, testosterone cypionate; TCsc, testosterone cypionate subcutaneous; TDP, testosterone in transdermal patch; TE, testosterone enanthate; TED, testosterone enanthate depot; TG, testosterone gel; TP, testosterone propionate; tT, total testosterone; TU, testosterone undecanoate.

### Quality assessment

The quality assessment based on the EPHPP is summarized in **Table 2**. Overall, most studies (15 of 20) received a methodological quality rating of ‘‘moderate’’ (12, 22, 24, 27–32, 34–37, 39, 40) and 5 studies were scored as ‘‘weak’’ (23, 25, 26, 33, 38). The items ‘‘confounders’’ and “data collection methods” received the highest rating among all the included studies; on the contrary, the item ‘‘blinding’’ was the most lacking, as in none of the studies the participants and the researchers who assessed outcomes were blind to the study conditions. Seven studies received a ‘‘moderate’’ or ‘‘weak’’ methodological quality rating in the item ‘‘withdrawals and dropouts’’ because of the large difference in the number of participants between initial enrollment and the end of follow-up (12, 23, 25, 26, 28, 33, 38).

**Table 2:** Quality assessment of included studies by Effective Public Health Practice Project quality assessment tool.

Study	Selection bias	Study design	Confounders	Blinding	Data collection methods	Withdrawals and drop-outs	Global rating
*Chen 2019* (22)	moderate	moderate	strong	weak	strong	strong	moderate
*Fadich 2022* (12)	moderate	moderate	strong	weak	strong	moderate	moderate
*Fernandez 2016* (23)	moderate	moderate	strong	weak	strong	weak	weak
*Humble 2019* (24)	moderate	moderate	strong	weak	strong	strong	moderate
*Kirisawa 2021* (25)	moderate	moderate	strong	weak	strong	weak	weak
*Korpaisarn 2021* (26)	moderate	moderate	strong	weak	strong	weak	weak
*Kurahashi 2013* (27)	moderate	moderate	strong	weak	strong	strong	moderate
*Liu 2022* (28)	moderate	moderate	strong	weak	strong	moderate	moderate
*Maheshwari 2022* (29)	moderate	moderate	strong	weak	strong	strong	moderate
*Meriggiola 2008* (30)	moderate	moderate	strong	weak	strong	strong	moderate
*Millington 2022* (31)	moderate	moderate	strong	weak	strong	strong	moderate
*Scharff 2019* (32)	moderate	moderate	strong	weak	strong	strong	moderate
*Stoffers 2019* (33)	moderate	moderate	strong	weak	strong	weak	weak
*Tominaga 2024* (34)	moderate	moderate	strong	weak	strong	strong	moderate
*van Eeghen 2023* (35)	moderate	moderate	strong	weak	strong	strong	moderate
*van Kesteren 1996* (36)	moderate	moderate	strong	weak	strong	strong	moderate
*Vlot 2019* (37)	moderate	moderate	strong	weak	strong	strong	moderate
*Wiepjes 2019* (38)	moderate	moderate	strong	weak	strong	weak	weak
*Wierckx 2014* (39)	moderate	moderate	strong	weak	strong	strong	moderate
*Yahyaoui 2008* (40)	moderate	moderate	strong	weak	strong	strong	moderate

### Primary outcome: glomerular filtration rate

**Figure 2:**
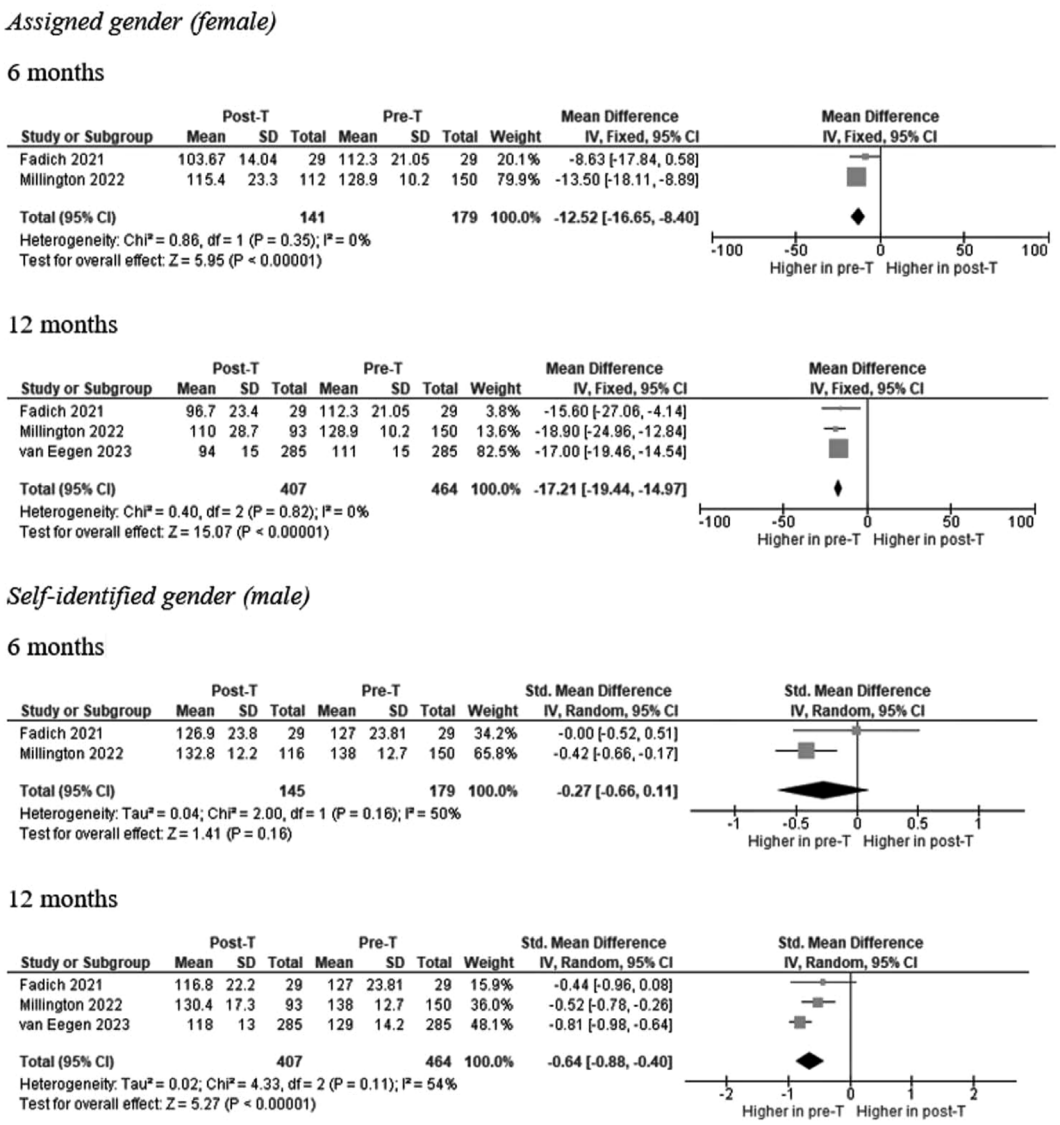
Forests plot of the effects of T-based GAHT on Chronic Kidney Disease Epidemiology Collaboration (CKD-EPI) mean values (ml/min/1.73 m²) in TM according to assigned **(A)** or perceived **(B)** gender. Diamonds indicate the overall effect estimates (and diamond width the 95% CI); squares indicate the weight of individual studies in the aggregate estimate. CI confidence interval, IV inverse variance, T Testosterone, GAHT gender affirming hormone therapy, TM transmen.

**Figure 3:**
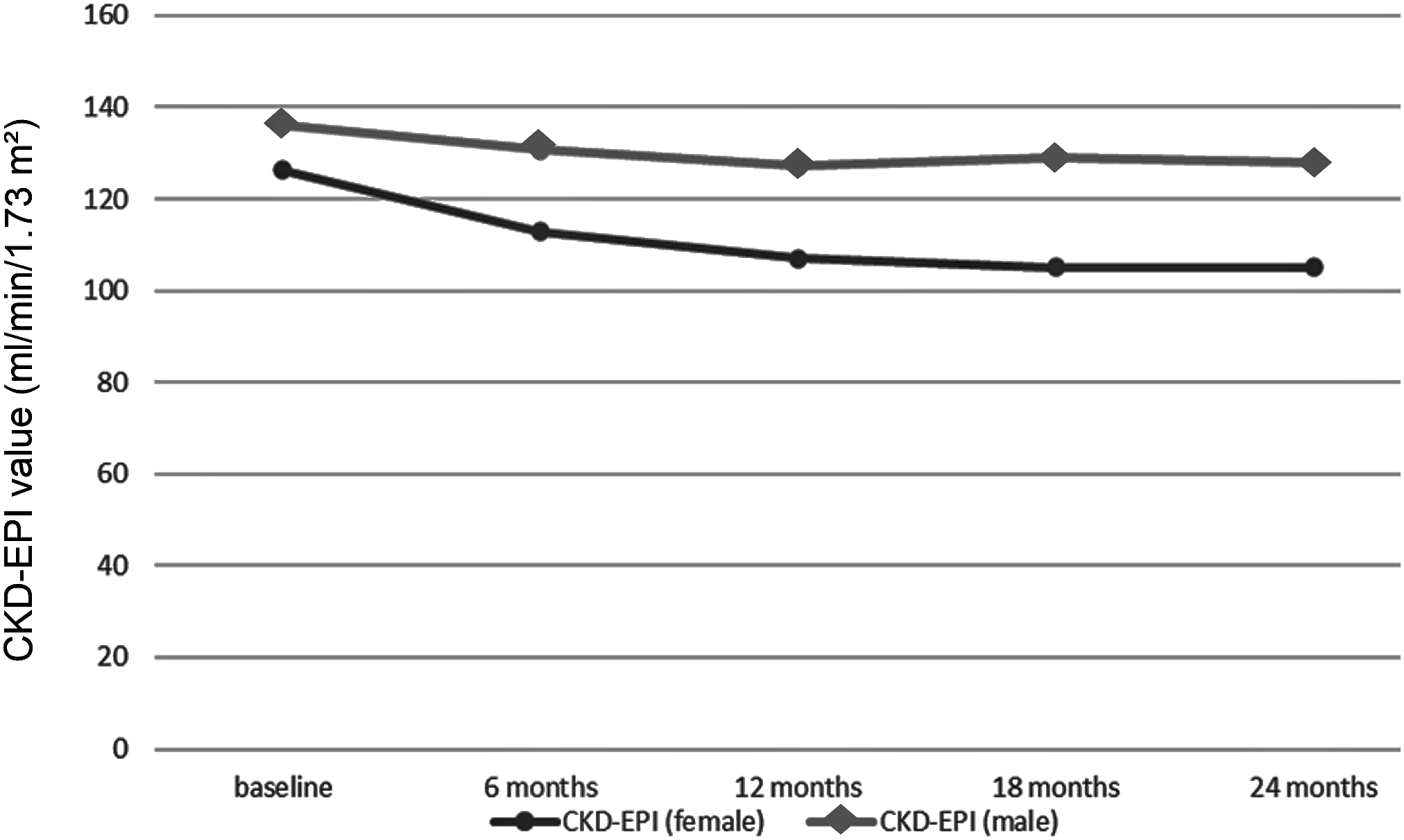
Trends in weighted average glomerular filtration rate values estimated using the Chronic Kidney Disease Epidemiology Collaboration (CKD-EPI) formula at each follow-up time point. The distinction between eGFR trajectories is represented using different markers: square symbols indicate estimates based on female sex in the CKD-EPI equation, while circles correspond to self-identified male gender.

Three studies reported information on calculated GFR in a total of 141 and 407 AFAB individuals after 6 and 12 months of T-GAHT, respectively (**Figure 2**). The overall mean difference (MD) documented a statistically significant decrease in GFR at 6 and 12 months as assessed by CKD-EPI equation, using the attributed (female) gender (6 months: MD = -12.52; 95% CI: -16.65, -8.4, p <0.0001; I^2^ = 0%, P_for heterogeneity_ = 0.35; 12 months: MD = -17.21; 95% CI: -19.44, -14.97, p <0.00001; I^2^ = 0%, P_for heterogeneity_ = 0.82). When the self-identified (male) gender was included in the CKD-EPI equation, a significant decrease in GFR was revealed after 12 months (MD = -0.64; 95% CI: -0.88, -0.40, p <0.00001; I^2^ = 54%, P_for heterogeneity_ = 0.11) but not 6 months of GAHT (MD = -0.27; 95% CI: -0.66, 0.11, p = 0.16; I^2^ = 50%, P_for heterogeneity_ = 0.35). The trend of the weighted averages of the estimated glomerular filtration rate values using the CKD-EPI formula according to attributed or self-identified gender at each follow-up time is presented in **Figure 3**: it suggests an initial decrease in eGFR during early T-GAHT, likely reflecting changes in creatinine production rather than impaired renal function.

### Secondary outcomes

Secondary endpoints included changes under GAHT in SCr, UA, BUN, SBP and DBP levels. Overall combined estimates documented a significant increase in SCr at all follow-up times (3, 6, 12, 18, and 24 months) compared with baseline, albeit with significant heterogeneity between studies after the third month (**Supplementary Figure 1**).

Overall, UA levels also increased significantly after 6, 12, and 24 months compared with baseline, with high reproducibility among studies (**Supplementary Figure 2**).

On the contrary, BUN did not change significantly after either 6 months or 12 months of therapy (**Supplementary Figure 3**).

Finally, while DBP did not change significantly at either 6 or 12 months (**Supplementary Figure 4A**), SBP increased significantly after 6 months of therapy, returning to values not significantly different from baseline after 12 months (**Supplementary Figure 4B**).

### Publication bias

Given the unavailability of an adequate number of studies for most of the outcomes analyzed, including the primary outcome, we assessed publication bias only for data on the change in SCrvalues at 12 months of therapy. As shown in **Supplementary Figure 5**, the asymmetric shape of the funnel plot might suggest the presence of publication bias. However, the trim-and-fill analysis did not identify any putative missing studies.

## Discussion

To our knowledge, this is the first systematic review and meta-analysis assessing the impact of testosterone-based gender affirming hormone therapy (T-GAHT) on kidney function in transgender individuals assigned female at birth (AFAB).

The direct effect of testosterone administration on kidney physiology has been explored in several preclinical studies using murine models, yielding inconclusive findings. Sex hormones appear to exert opposing actions on renal tissue: testosterone induces podocyte injury, while estrogens are protective (41–43). Conversely, in male rats, testosterone has been shown to mitigate renal ischemia–reperfusion injury, independently of estradiol (44). Lichtenecker et al. reported that testosterone treatment in female rats increased glomerular area and kidney size after four months, although this was associated with reduced GFR and histological changes (45). Other studies in male rodents have shown that early orchiectomy may prevent proteinuria and delay glomerulosclerosis (46), suggesting a detrimental role of androgens. In humans, androgens have been implicated in increased blood pressure and impaired renal function (47, 48), potentially through enhanced tubular sodium and water reabsorption (49) and activation of vasoconstrictive pathways such as the renin–angiotensin system and endothelin (48, 50–52). In line with these findings, our analysis showed a transient increase in systolic blood pressure after six months of T-GAHT, followed by stabilization.

In recent years, several studies have examined how best to estimate GFR in transgender individuals receiving hormone therapy (12, 31, 35), including whether to use the sex assigned at birth or the individual’s self-identified gender. In our analysis, we assessed changes in eGFR using both approaches across all follow-up timepoints. As highlighted by Krasowski, this choice impacts CKD stage classification according to KDIGO 2013 guidelines (53). Most of the included studies relied on the 2009 version of the CKD-EPI equation, which incorporates a race-based correction. Although the updated 2021 race-neutral equation (54) is now recommended, particularly in the United States (55), we used the published eGFR values without recalculating them due to the lack of access to individual-level data. This is acknowledged as a methodological limitation.

Using the female coefficient in CKD-EPI, despite ongoing masculinization and male gender identification, may overestimate renal function during follow-up. This explains the artifactual increase in eGFR observed when switching from sex-assigned-at-birth to affirmed-gender coefficients (**Figure 3**). However, when a consistent coefficient is applied over time, eGFR values decrease at 6 and 12 months and then stabilize at 18 and 24 months.

Whether this pattern reflects true renal impairment remains uncertain. The observed rise in SCr during the first year of T-GAHT, as reported in several studies (56, 57), likely reflects testosterone-induced increases in muscle mass and altered body composition (58, 59). This interpretation is supported by the concomitant rise in uric acid levels, which plateau after 12 months (**Supplementary Figure 2**), consistent with increased purine metabolism.

Transient hemodynamic changes, such as the rise in systolic blood pressure at six months (**Supplementary Figure 4B**), may also contribute to these changes. However, the absence of significant alterations in BUN (**Supplementary Figure 3**) and the lack of reported cases of new-onset hypertension or antihypertensive treatment suggest that these variations are more likely to represent a physiological adaptation to masculinization rather than early signs of pathological renal involvement (60, 61), consistent with recent insights into testosterone’s cardiovascular effects during gender-affirming therapy (62). Future research should clarify whether T-GAHT poses any clinically significant risk of hypertension.

This meta-analysis has several limitations. The observational nature of all included studies, combined with the absence of control groups, limits the ability to control for confounding variables. Attrition bias is also notable, as several studies reported substantial loss to follow-up. Moreover, most participants were young and had normal kidney function at baseline, which restricts the generalizability of our findings to older individuals or those with pre-existing renal disease. Additionally, none of the included studies assessed alternative biomarkers of renal function, such as cystatin C, which is less influenced by muscle mass and may better reflect glomerular filtration in individuals undergoing masculinizing hormone therapy. Although van Eeghen et al. (35) acknowledged the potential value of cystatin C, no longitudinal data were reported. Finally, while a few studies mentioned the concurrent use of GnRH agonists (30, 33), none stratified renal outcomes by treatment regimen or explored the specific impact of GnRH suppression. This remains an important area for future investigation.

In conclusion, testosterone-based GAHT appears to have a statistically significant impact on eGFR in healthy, young AFAB individuals during the first two years of therapy. However, these changes are unlikely to reflect clinically meaningful renal impairment. eGFR values—regardless of whether calculated using male or female CKD-EPI coefficients—tend to stabilize after 12 months and remain well above thresholds for renal dysfunction. These findings underscore the importance of cautious interpretation of renal function markers during early T-GAHT. Further studies are needed to evaluate these effects in more diverse populations, particularly in older individuals and those with existing kidney disease, and to validate sex-independent tools for accurately monitoring renal function in transgender people receiving gender-affirming hormone therapy.

## Data Availability

The original contributions presented in the study are included in the article/**Supplementary Material**. Further inquiries can be directed to the corresponding author.

## Supplementary material

Supplementary Figure 1 Forest plot of the effects of T-based GAHT on creatinine mean values (mg/dl) in TM. Diamonds indicate the overall effect estimates (and diamond width the 95% CI); squares indicate the weight of individual studies in the aggregate estimate. CI confidence interval, IV inverse variance, T Testosterone, GAHT gender affirming hormone therapy, TM transmen.

Supplementary Figure 2 Forest plot of the effects of T-based GAHT on uric acid values (mg/dl) in TM. Diamonds indicate the overall effect estimates (and diamond width the 95% CI); squares indicate the weight of individual studies in the aggregate estimate. CI confidence interval, IV inverse variance, T Testosterone, GAHT gender affirming hormone therapy, TM transmen.

Supplementary Figure 3 Forest plot of the effects of T-based GAHT on BUN values in TM. Diamonds indicate the overall effect estimates (and diamond width the 95% CI); squares indicate the weight of individual studies in the aggregate estimate. BUN blood urea nitrogen (mg/dl), CI confidence interval, IV inverse variance, T Testosterone, GAHT gender affirming hormone therapy, TM transmen.

Supplementary Figure 4 Forest plot of the effects of T-based GAHT in TM on diastolic (**A**) and systolic (**B**) blood pressure values (mmHg). Diamonds indicate the overall effect estimates (and diamond width the 95% CI); squares indicate the weight of individual studies in the aggregate estimate. CI confidence interval, IV inverse variance, T Testosterone, GAHT gender affirming hormone therapy, TM transmen.

Supplementary Figure 5 Funnel plot of results from studies assessing changes in creatinine levels (mg/dl) after 12 months of testosterone-based gender affirming hormone therapy.
