# Supplementary figures and images for: Effects of gender affirming hormone therapy with testosterone on renal function of assigned female at birth transgender people: a meta-analysis
Source: Front Endocrinol (Lausanne). 2025 Jun 12;16:1537838. doi: 10.3389/fendo.2025.1537838 (PMC12198172; doi:10.3389/fendo.2025.1537838)

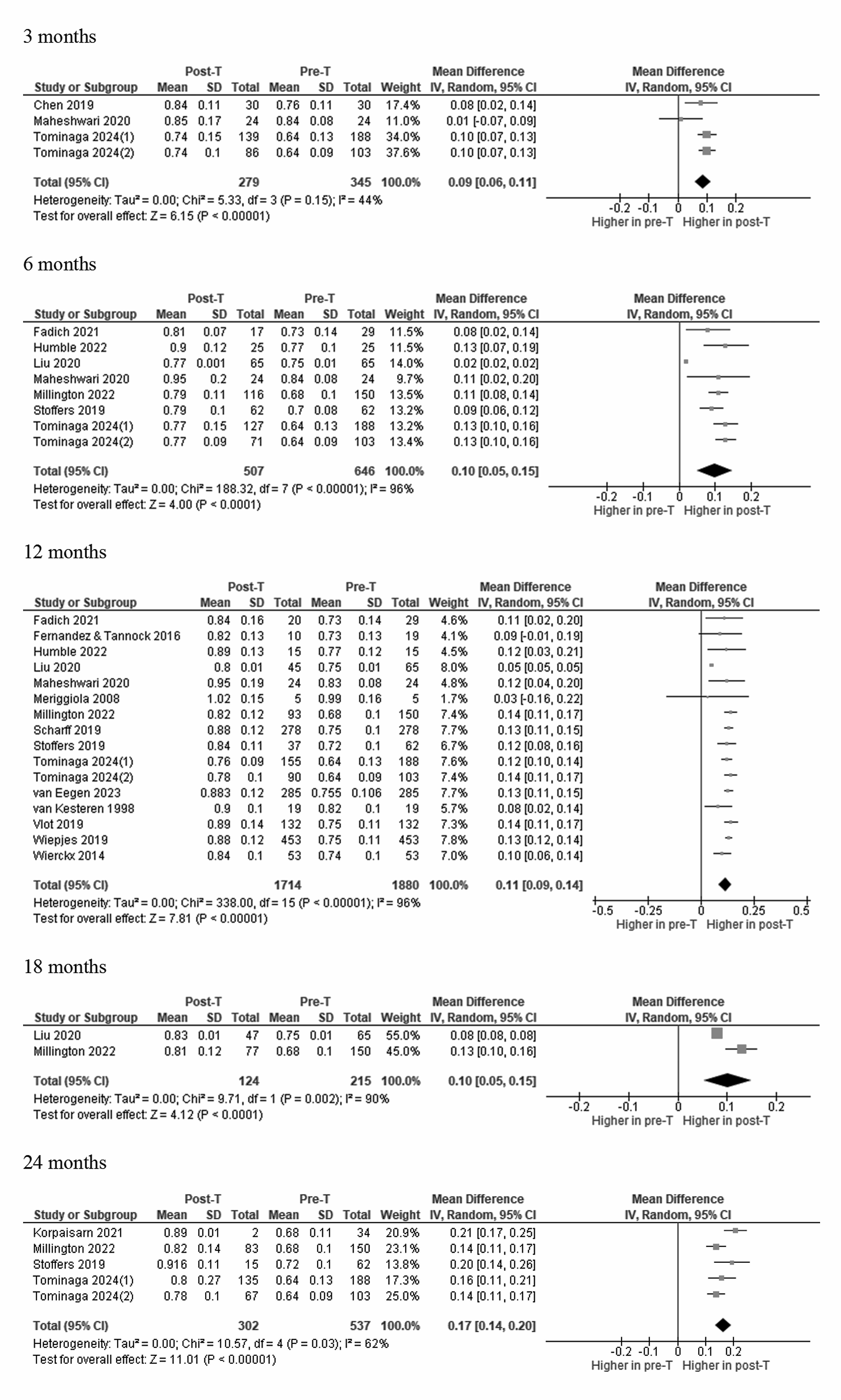

Supplement: Supplementary Figure 1 — Forest plot of the effects of T-based GAHT on creatinine mean values (mg/dl) in TM. Diamonds indicate the overall effect estimates (and diamond width the 95% CI); squares indicate the weight of individual studies in the aggregate estimate. CI confidence interval, IV inverse variance, T Testosterone, GAHT gender affirming hormone therapy, TM transmen. [file Image1.tif]

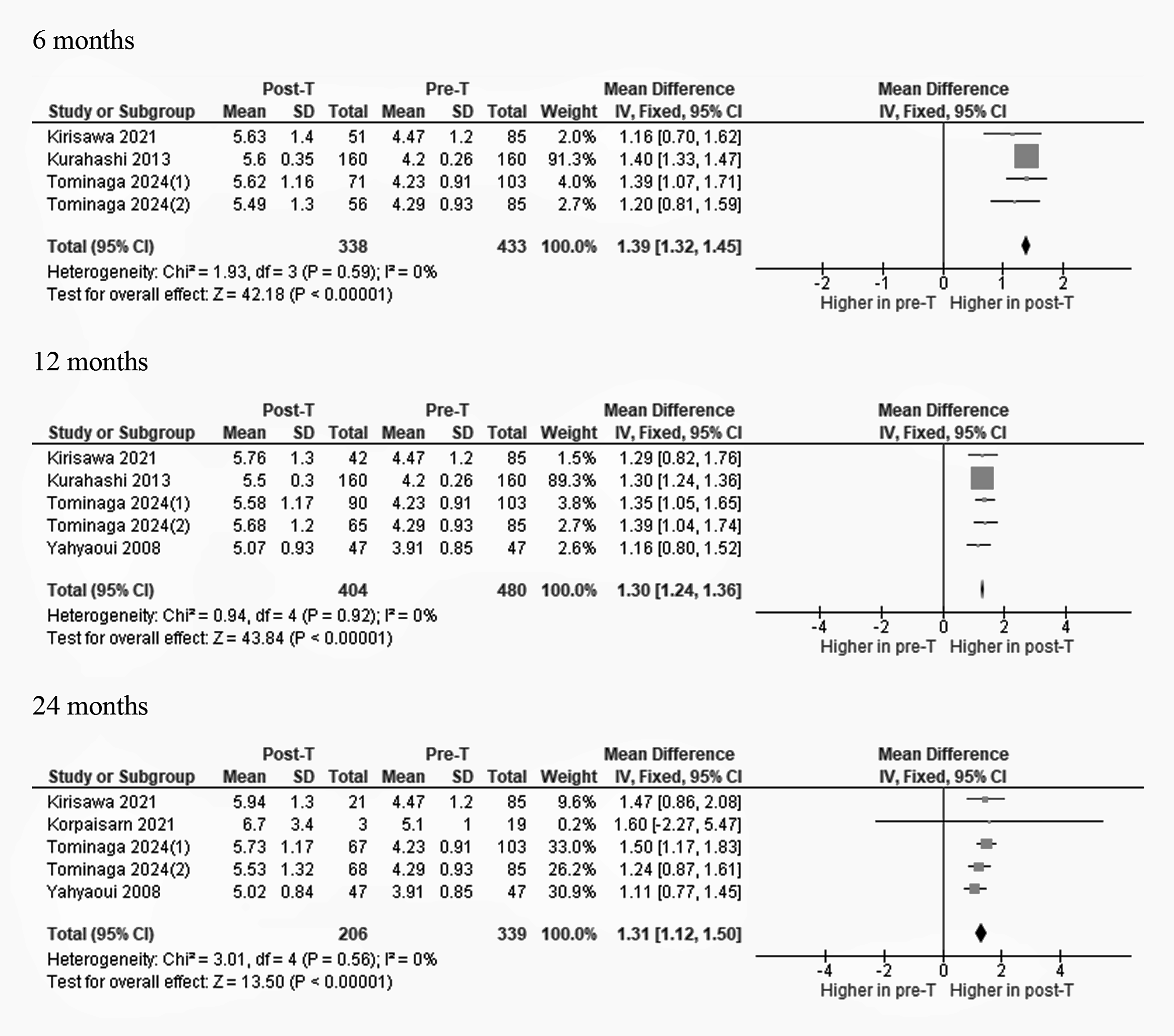

Supplement: Supplementary Figure 2 — Forest plot of the effects of T-based GAHT on uric acid values (mg/dl) in TM. Diamonds indicate the overall effect estimates (and diamond width the 95% CI); squares indicate the weight of individual studies in the aggregate estimate. CI confidence interval, IV inverse variance, T Testosterone, GAHT gender affirming hormone therapy, TM transmen. [file Image2.tif]

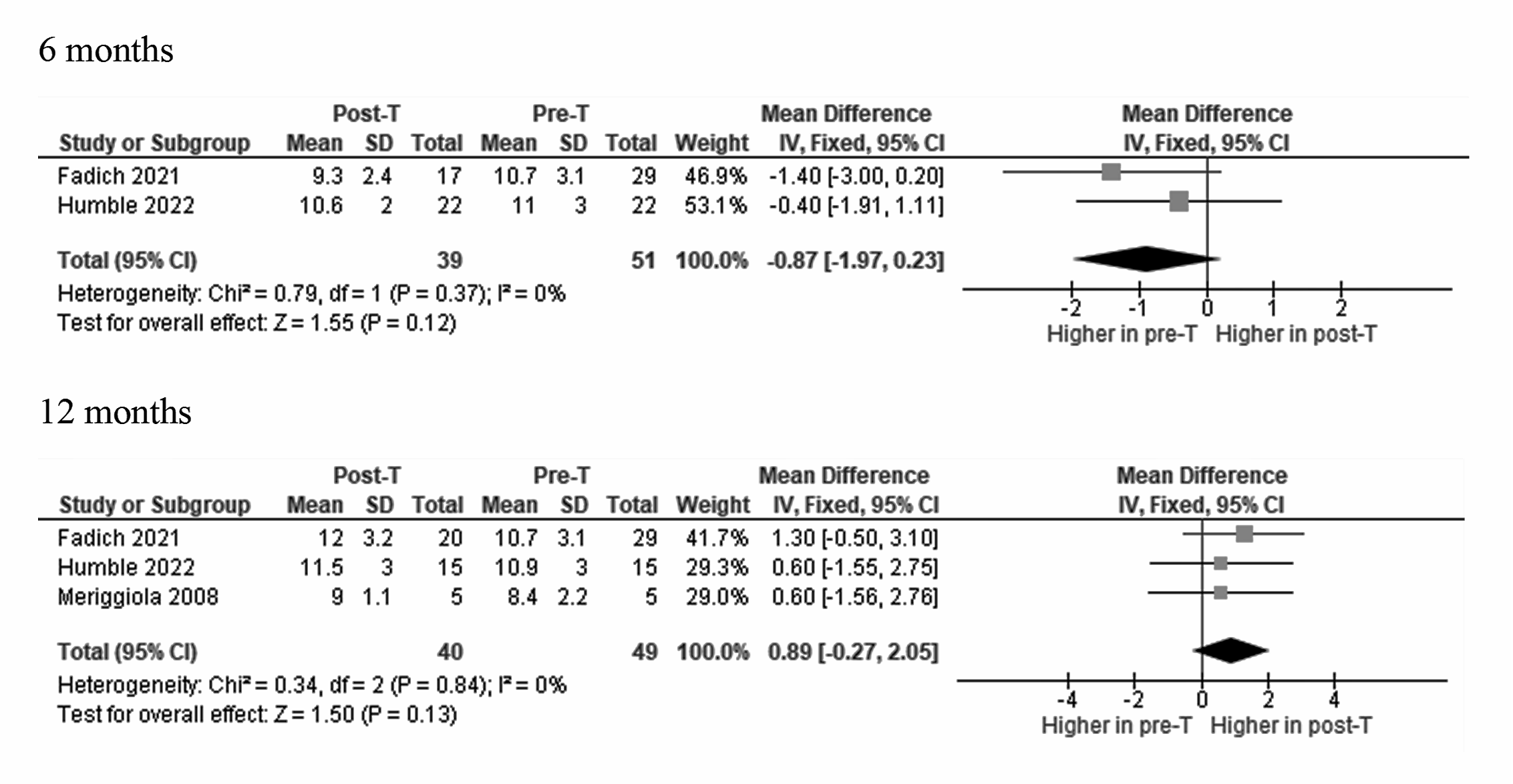

Supplement: Supplementary Figure 3 — Forest plot of the effects of T-based GAHT on BUN values in TM. Diamonds indicate the overall effect estimates (and diamond width the 95% CI); squares indicate the weight of individual studies in the aggregate estimate. BUN blood urea nitrogen (mg/dl), CI confidence interval, IV inverse variance, T Testosterone, GAHT gender affirming hormone therapy, TM transmen. [file Image3.tif]

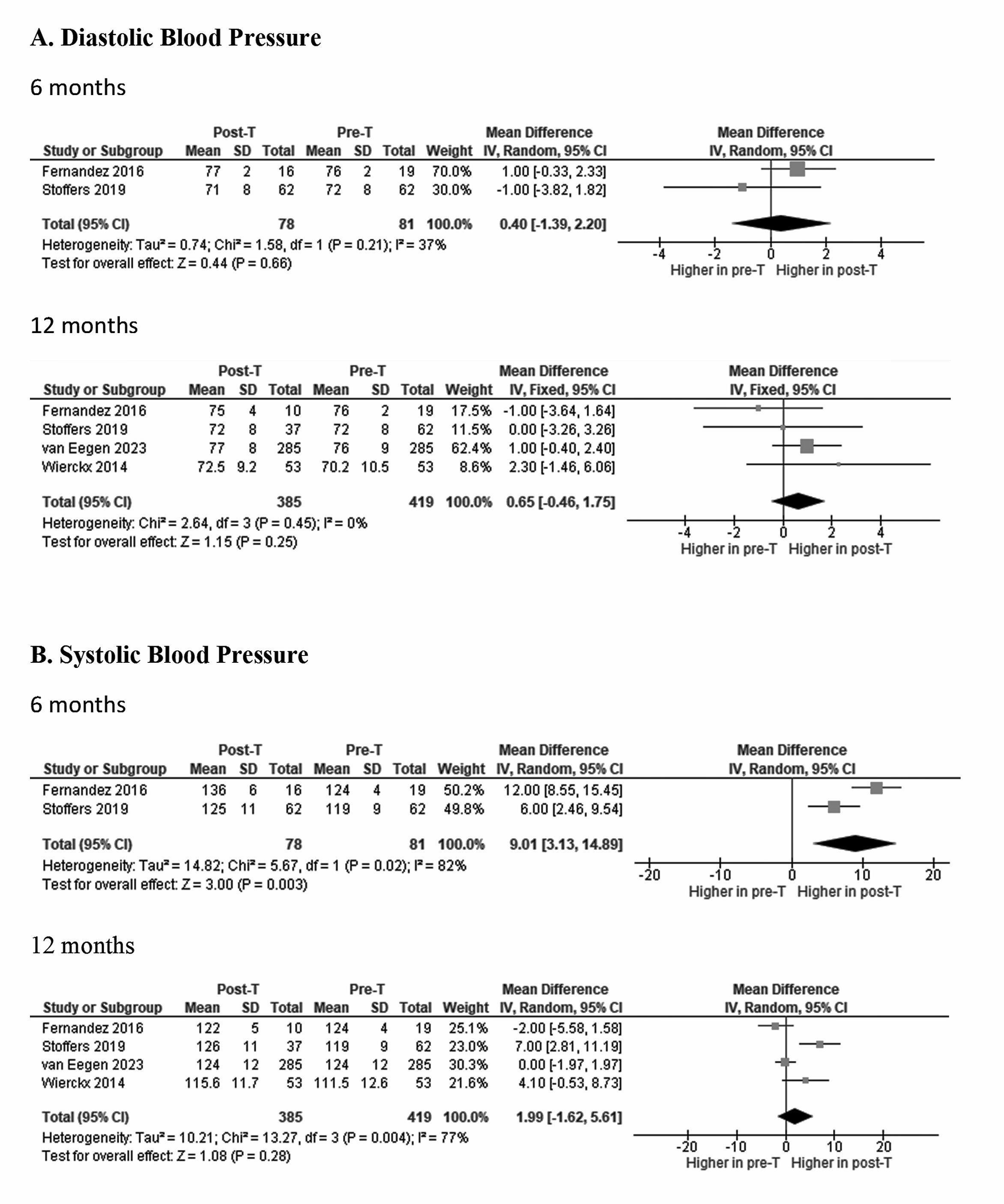

Supplement: Supplementary Figure 4 — Forest plot of the effects of T-based GAHT in TM on diastolic (A) and systolic (B) blood pressure values (mmHg). Diamonds indicate the overall effect estimates (and diamond width the 95% CI); squares indicate the weight of individual studies in the aggregate estimate. CI confidence interval, IV inverse variance, T Testosterone, GAHT gender affirming hormone therapy, TM transmen. [file Image4.tif]

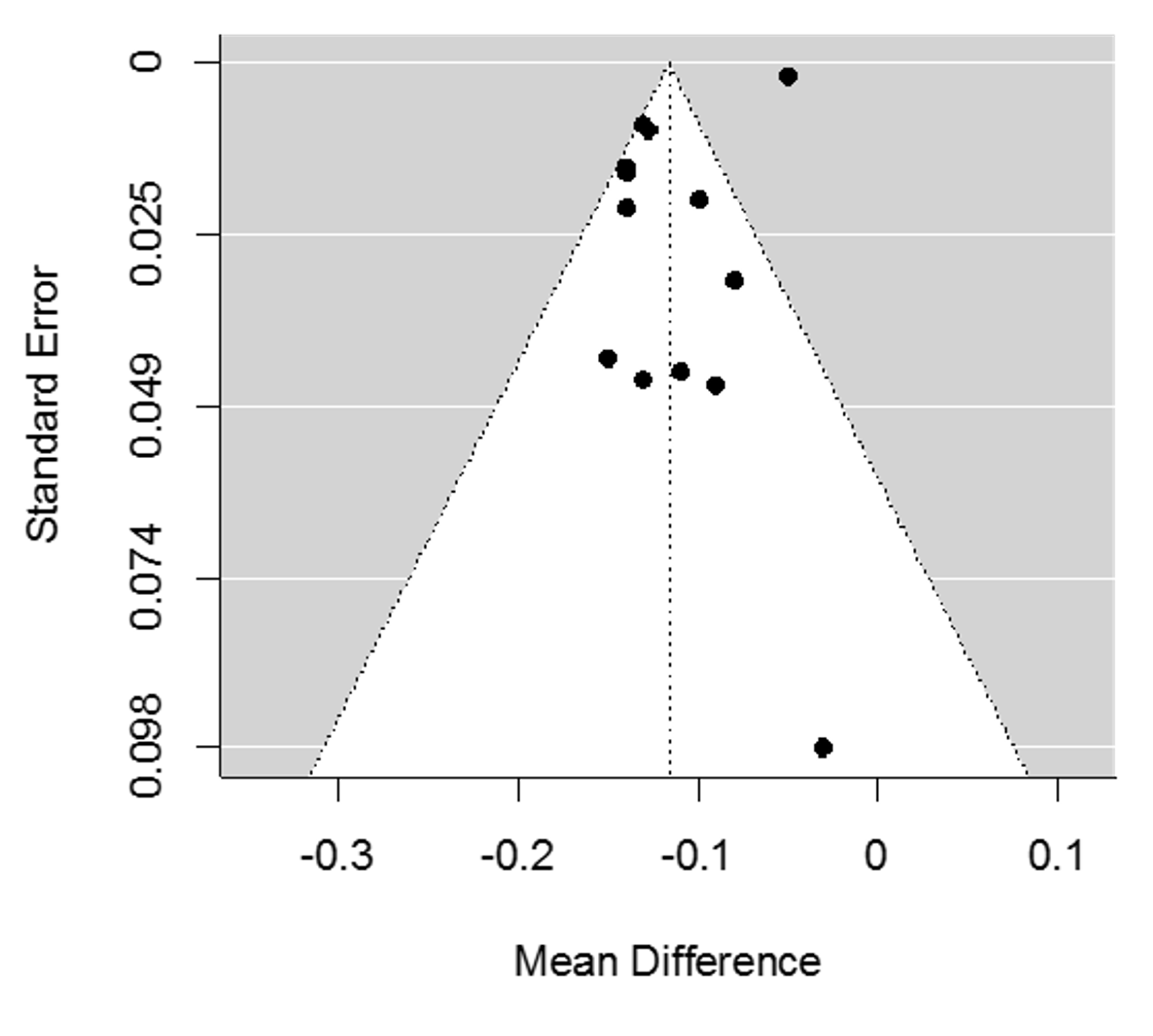

Supplement: Supplementary Figure 5 — Funnel plot of results from studies assessing changes in creatinine levels (mg/dl) after 12 months of testosterone-based gender affirming hormone therapy. [file Image5.tif]
